# Non-Small-Cell Lung Cancer: From Histopathological Classification to Precision Oncology—A Narrative Review

**DOI:** 10.3390/jcm15083042

**Published:** 2026-04-16

**Authors:** Simona-Maria Roșu, Viorel Biciușcă, Sorina-Ionelia Stan, Denisa Maria Mitroi, Beatrice Mahler, Diana-Maria Trașcă, Mihaela Popescu, Marian-Marius Pădureanu, Ana-Ștefania Stoica, Tania-Ioana Pencea, Ionela-Alina Croitoru, Mara Amalia Bălteanu

**Affiliations:** 1 Doctoral School, University of Medicine and Pharmacy of Craiova, 200349 Craiova, Romania; denisa_maria2@yahoo.com (D.M.M.); marius.padureanu98@yahoo.com (M.-M.P.); stoicaanastefania@gmail.com (A.-Ș.S.); taniagoncea@yahoo.com (T.-I.P.); 2 Department of Internal Medicine, Faculty of Medicine, University of Medicine and Pharmacy of Craiova, 200349 Craiova, Romania; sorina_stan@icloud.com (S.-I.S.); diana.trasca@umfcv.ro (D.-M.T.); 3 Department of Pneumology, “Marius Nasta” Institute for Pneumology, 050159 Bucharest, Romania; beatrice.mahler@umfcd.ro (B.M.); alinahaulinca@umfcd.ro (I.-A.C.); mara.balteanu@prof.utm.ro (M.A.B.); 4 Department of Pneumology, Faculty of Medicine, University of Medicine and Pharmacy Carol Davila of Bucharest, 020021 Bucharest, Romania; 5 Department of Endocrinology, Faculty of Medicine, University of Medicine and Pharmacy of Craiova, 200349 Craiova, Romania; mihaela.popescu.e@umfcv.ro; 6 Department of Pneumology, Faculty of Medicine, “Titu Maiorescu” University, 040051 Bucharest, Romania

**Keywords:** non-small-cell lung cancer, histological subtypes, actionable oncogenic drivers, treatment, multidisciplinary management

## Abstract

Non-Small-Cell Lung Cancer (NSCLC) represents the most prevalent form of lung cancer and remains one of the leading causes of cancer-related morbidity and mortality worldwide. This disease has evolved far beyond traditional histopathological classification. While histology remains foundational, it is no longer sufficient to guide optimal patient management in the era of precision oncology. This review uniquely integrates the full spectrum of NSCLC evaluation, from underlying pathophysiological mechanisms to histological, immunohistochemical, and molecular analyses, culminating in individualized therapeutic planning. We highlight actionable genetic alterations—including EGFR, ALK, ROS1, BRAF, and KRAS—and their roles in guiding targeted therapies, alongside the transformative impact of immune checkpoint inhibitors in selected patients. By emphasizing the interplay between tumor biology, diagnostic workflows, and treatment selection, this review underscores the necessity of comprehensive molecular testing and data integration. Finally, we discuss emerging biomarkers and rational combination strategies that promise to further refine patient stratification and improve outcomes.

## 1. Introduction

NSCLC represents the most common form of lung cancer and exhibits significant histological, molecular, and clinical heterogeneity. Historically, NSCLC classification relied primarily on morphological assessment of tumor tissue, distinguishing major subtypes such as adenocarcinoma, squamous cell carcinoma, and large cell carcinoma. While these histopathological distinctions provided a foundation for prognosis and therapeutic decision-making, they were insufficient to guide personalized treatment in the era of precision oncology. Over the past decade, advances in molecular biology and immunohistochemistry have transformed the diagnostic and therapeutic landscape of NSCLC [1]. The identification of actionable oncogenic drivers—including EGFR mutations, ALK and ROS1 rearrangements, BRAF alterations, and KRAS mutations—has enabled targeted therapies that achieve superior response rates and improved survival [2]. Simultaneously, the assessment of PD-L1 expression and other immune biomarkers has facilitated patient selection for immunotherapy, providing durable responses in selected populations [3]. These developments have shifted the focus from morphology alone to an integrated approach in which histology, immunohistochemistry, and molecular profiling collectively inform treatment strategies [4]. This review aims to provide a contemporary perspective on NSCLC by emphasizing how traditional histopathological classification has evolved in the context of modern precision medicine. We discuss which histological distinctions remain clinically meaningful, how immunohistochemical markers complement morphological evaluation, and how comprehensive genomic profiling guides targeted and immunotherapeutic interventions. By framing NSCLC management within this integrated paradigm, the review highlights the convergence of morphology and molecular biology in current clinical practice, illustrating the transition from descriptive classification to biomarker-driven precision oncology.

## 2. Epidemiology

### 2.1. General Data

**Figure 1:**
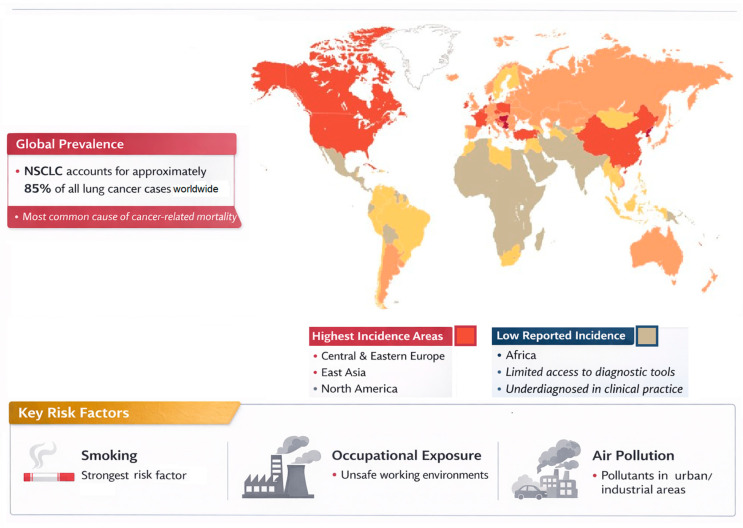
Epidemiology of Non-Small-Cell Lung Cancer.

NSCLC is the most common subtype of lung cancer globally and remains a major contributor to cancer deaths. Despite advances in screening and prevention programs, its incidence continues to be elevated in both high- and low-income regions. In countries that have implemented strong smoking control strategies, a slow but steady decline in overall lung cancer rates has been observed. Even so, this positive trend is tempered by the increasing proportion of adenocarcinoma cases. In particular, the increasing incidence of adenocarcinoma among women and non-smokers underscores changing risk patterns and highlights the increasingly complex epidemiology of this lung cancer subtype. NSCLC accounts for approximately 85% of all reported cases, and the burden of the disease is not evenly distributed worldwide [5]. The highest incidence rates are consistently documented in regions such as Central and Eastern Europe, East Asia, and North America. In addition, several areas in Africa appear to have lower reported rates, a pattern that is often attributed to limited access to diagnostic tools and underdiagnosis of the disease in clinical practice. Socioeconomic conditions also play a critical role in shaping the prevalence of NSCLC [6]. Communities that experience economic hardship tend to be more exposed to major risk factors—including higher rates of smoking, unsafe occupational environments, and high levels of environmental pollutants (Figure 1). This underscores the importance of both public health interventions and improved access to medical infrastructure [7].

### 2.2. Risk Factors

Smoking continues to be the dominant risk factor for NSCLC, with most cases caused by active smoking, as well as prolonged exposure to tobacco smoke—passive smoking [7]. Several environmental and occupational factors also significantly increase the likelihood of developing NSCLC. Prolonged exposure to radon, asbestos fibers, heavy metals, and polycyclic aromatic hydrocarbons plays an important role in lung carcinogenesis, particularly among people working in industries such as mining, construction, and metallurgy [8]. Air pollution has emerged as a critical factor contributing to the risk of lung cancer. Fine particles, particularly PM2.5, have been strongly associated with an increased incidence of NSCLC in heavily industrialized or densely populated urban areas [9]. Although NSCLC is primarily an acquired disease, genetic factors may influence individual susceptibility. A positive family history may indicate an inherited predisposition, and even though true hereditary mutations are uncommon, certain germline genetic variants may alter the body’s response to carcinogens and therefore increase overall risk [10]. Additionally, certain pulmonary diseases may act as risk factors for lung cancer. A history of pulmonary tuberculosis may increase this risk through persistent infection with Mycobacterium tuberculosis, which promotes chronic inflammation, fibrosis, and parenchymal scarring, creating a microenvironment that may favor neoplastic development [11]. This chronic inflammatory process is also characteristic of chronic obstructive pulmonary disease, which likewise represents an important risk factor for the development of lung cancer [12].

### 2.3. Sex and Age Distribution

Epidemiological data show a steady increase in the number of cases among women, although NSCLC is still more common in men. This trend is associated both with the increase in the incidence of smoking among women in recent decades and with specific biological characteristics that influence individual susceptibility to the disease [13]. Adenocarcinoma is considered particularly common in women and in people who have never smoked [14]. The incidence of NSCLC increases considerably after the age of 50 and reaches its highest levels in the sixth and seventh decades of life. However, in recent years, an increasing number of cases have been reported in younger patients. Although less common, these manifestations are frequently linked to well-defined oncogenic mutations, such as alterations in the EGFR, ALK, or ROS1 genes, which can influence both tumor behavior and response to targeted therapies [15].

## 3. Pathogenesis

The development of NSCLC is driven by a complex interplay between genetic predisposition, epigenetic modifications, and environmental exposures. All these factors lead to the gradual transformation of healthy respiratory epithelial cells into malignant tissue [16]. The process unfolds over time as key molecular abnormalities accumulate, disrupting normal regulatory pathways that govern cell division, differentiation, and programmed cell death [17].

### 3.1. General Mechanisms

NSCLC develops through the gradual transformation of normal respiratory epithelial cells into malignant tissue, driven by the interplay of genetic predisposition, epigenetic changes, and environmental exposures. Key molecular abnormalities disrupt cell cycle regulation, apoptosis, and immune surveillance, enabling sustained proliferation and survival of abnormal cells. Chronic irritation from tobacco smoke or air pollutants promotes inflammation and oxidative stress, further damaging DNA and fostering genomic instability [18,19]. Aberrant activation of signaling pathways, such as PI3K/AKT, RAS/RAF/MEK, and JAK/STAT, enhances tumor growth, angiogenesis, and invasiveness, laying the foundation for NSCLC progression and metastasis [20].

### 3.2. Specific Genetic Alterations

Each histological subtype of NSCLC displays a unique molecular signature that influences both tumor behavior and therapeutic response. Adenocarcinoma is frequently associated with activating mutations in EGFR, rearrangements in ALK and ROS1, and mutations in KRAS and BRAF. These alterations result in abnormal activation of signaling pathways such as tyrosine kinase receptors or G-protein-coupled cascades, promoting sustained proliferation, evasion of apoptosis, and enhanced survival [21]. Squamous cell carcinoma is commonly linked to TP53 mutations, impairing DNA damage repair, and FGFR1 amplifications, which promote tumor growth and survival [22]. Large cell carcinoma is molecularly heterogeneous, with no single predominant driver mutation, and is often a diagnosis of exclusion after other NSCLC subtypes are ruled out [23]. Importantly, these molecular alterations are not only involved in tumorigenesis but also represent clinically actionable targets. The identification of EGFR mutations, ALK or ROS1 rearrangements, and BRAF and KRAS alterations enables the use of targeted therapies, such as tyrosine kinase inhibitors and BRAF/MEK inhibitors, which have demonstrated superior response rates, improved survival, and effective control of both systemic and intracranial disease. This therapeutic relevance underscores the necessity of comprehensive molecular profiling in modern NSCLC management, bridging traditional histopathology with precision oncology [24].

### 3.3. Mechanisms of Tumor Progression and Metastasis

Profound remodeling of the tumor microenvironment through the gradual accumulation of genetic alterations leads to NSCLC progression, allowing tumor cells to grow abnormally, resist apoptosis, and acquire invasive properties. Epithelial–mesenchymal transition is the mechanism by which tumor cells become mesenchymal and can invade surrounding tissues and disseminate distantly. The release of vascular endothelial growth factor leads to angiogenesis and neovascularization at the tumor level, which provides essential nutrients and oxygen to support tumor growth and constitutes an access route for cancer cells to enter the circulation [25]. Once in the blood or lymphatic system, tumor cells disseminate and can form secondary tumors in the brain, bones, liver, adrenal glands, and the contralateral lung. Secondary cardiac metastases in NSCLC are frequently underdiagnosed despite their significant clinical impact. They can arise via direct invasion, hematogenous spread, or lymphatic dissemination, affecting the pericardium, myocardium, and endocardium, and are often overlooked by clinicians because symptoms—such as dyspnea, palpitations, arrhythmias, chest pain, or signs of heart failure—are nonspecific and may be attributed to systemic tumor progression or pre-existing cardiovascular comorbidities. Early detection is crucial, as cardiac involvement has direct implications for treatment planning and prognosis: the presence of cardiac lesions may influence decisions regarding chemotherapy, radiotherapy, or targeted therapies and may necessitate closer cardiac monitoring [26]. Together, all these mechanisms underscore the aggressive and adaptive nature of NSCLC, highlighting the challenges in controlling tumor progression and preventing metastasis [27].

## 4. Pathological Features

The pathological features of NSCLC constitute a fundamental component of diagnostic evaluation and play a decisive role in prognosis and therapeutic planning. A thorough pathological evaluation integrates macroscopic appearance, detailed microscopic examination, immunohistochemical profiling, and molecular characterization. These features allow for the precise determination of tumor subtype, biological behavior, and characteristics that predict recurrence, progression, or metastatic spread [28].

### 4.1. General Macroscopic and Microscopic Characteristics

From a macroscopic perspective, NSCLC demonstrates considerable heterogeneity, influenced primarily by tumor histology and anatomical site of origin. Centrally located tumors usually arise from the larger bronchi and may obstruct the airway, while peripheral lesions typically develop within the distal lung parenchyma, sometimes in association with areas of fibrosis or chronic inflammatory remodeling [29]. Adenocarcinoma, the most prevalent subtype, frequently presents as a peripheral nodule. These tumors may appear solid, partially solid, or with regions of parenchymal consolidation. Some lesions exhibit a desmoplastic reaction or are associated with pre-existing interstitial scarring, reflecting their origin from alveolar epithelial cells [30]. Squamous cell carcinoma commonly develops in the central airways. Macroscopically, it often forms bulky, firm masses capable of invading and distorting the bronchial lumen. Areas of necrosis and cavitation are frequent due to impaired vascular supply within the tumor core [31]. Large cell carcinoma usually manifests as a well-circumscribed mass with a soft, friable texture. Pronounced necrosis is a typical finding, consistent with its high-grade, poorly differentiated nature [32]. Microscopically, adenocarcinomas display a spectrum of architectural patterns—lepidic, acinar, papillary, micropapillary, and solid. These patterns are not merely descriptive but carry prognostic weight: for example, micropapillary and solid components are associated with increased aggressiveness and poorer clinical outcomes [33]. Squamous cell carcinomas are characterized by unequivocal squamous differentiation, evidenced by keratinization, formation of keratin pearls, and identifiable intercellular bridges. These features help distinguish them from other epithelial malignancies [34]. Large cell carcinoma is diagnosed largely by exclusion [35]. These tumors lack glandular or squamous differentiation, both morphologically and immunohistochemically and represent a poorly differentiated, high-grade neoplasm.

### 4.2. Invasion Patterns and Pathological Staging Criteria (TNM)

Assessment of tumor invasion into adjacent anatomical structures is a central component of the pathological evaluation of NSCLC. As the tumor grows, it may infiltrate the bronchial tree, compromise the pulmonary vasculature, or penetrate the visceral and parietal pleura. More advanced lesions may extend beyond the lung into the chest wall, diaphragm, pericardium, or mediastinum, and such local invasion is associated with an advanced stage and a poor prognosis [36]. Lung cancer staging is based on the internationally standardized Tumor-Node-Metastasis (TNM) staging system, the 9th version of which was last published in 2025. The T category refers to the maximum size of the primary lesion, its extent, and the presence of pleural, vascular, or airway invasion. The N category assesses regional lymph node involvement. The number, size, and anatomical location of involved nodes—especially spread to mediastinal or subcarinal sites—have major prognostic implications and frequently influence the therapeutic strategy. The M category documents the presence of distant spread, either to the contralateral lung, pleura, or to the bone, liver, brain, or other organs [37]. Accurate staging requires meticulous examination of resected specimens, including systematic lymph node dissection or sampling, careful assessment of surgical margins, and detailed histological evaluation of the routes of invasion. This approach ensures appropriate classification, facilitates individualized treatment planning, and ultimately contributes to improved prognostic accuracy.

### 4.3. Predictive and Prognostic Factors

In addition to the parameters defined by the TNM classification, multiple additional pathological features significantly influence the course of NSCLC. Thus, blood and lymphatic vessel invasion stands out as one of the strongest predictors of an unfavorable prognosis, as this fact reflects an increased capacity for systemic dissemination and is associated with both early recurrence and distant metastasis [38]. The degree of cellular differentiation also provides important prognostic information. Well-differentiated tumors generally show slower growth and a more favorable course, compared with poorly differentiated or undifferentiated neoplasms that tend to have rapid progression, increased invasive potential, and a higher probability of resistance to treatment. Tumor necrosis, which often reflects hypoxia in rapidly proliferating lesions, is frequently associated with aggressive behavior. The density and composition of inflammatory infiltrates—including lymphocytes, macrophages, and stromal cells—may indicate an altered immune microenvironment that supports tumor progression [39]. Additionally, predictive factors in NSCLC have increasingly shifted from purely morphological criteria toward molecularly defined subgroups. Driver mutations (EGFR, ALK, ROS1, BRAF, KRAS) and PD-L1 expression not only influence prognosis but are critical for therapeutic decision-making, guiding the selection of tyrosine kinase inhibitors, immune checkpoint inhibitors, and combination therapies. In current practice, histopathological classification alone is insufficient for optimal patient management. Accurate subtyping is achieved through a combination of histology, immunohistochemical markers, and comprehensive molecular profiling, ensuring that each patient is matched with the most effective targeted or immunotherapeutic approach. This integrative strategy bridges traditional morphology with precision oncology, enabling both prognostic assessment and predictive therapeutic selection.

## 5. Clinical Manifestations

The clinical presentation of NSCLC includes multiple signs and symptoms, depending on the location, size, growth rate, and local or distant spread of the tumor. In the early stages, it does not produce obvious symptoms, allowing the disease to progress silently. Therefore, a significant percentage of cases are identified incidentally during routine imaging evaluations, such as preoperative imaging or cardiovascular investigations. When symptoms occur, they usually correlate with disruption of normal lung structure and function. Tumors located centrally in the main bronchi or proximal airways are more likely to cause early respiratory symptoms. Symptoms may include persistent cough, dyspnea, wheezing, or hemoptysis due to airway obstruction, mucosal irritation, or erosion of vascular structures. Peripheral tumors, on the other hand, may remain clinically silent until they reach a considerable size or invade the pleura, at which point patients may develop chest pain or signs of pleural effusion [40]. Systemic manifestations usually accompany more advanced disease and are often the result of tumor-induced metabolic derangements, chronic inflammation, or cytokine release, and include fatigue, anorexia, weight loss, and generalized decline in functional status. Some patients may present with manifestations of paraneoplastic syndromes that signify ectopic hormone secretion or immune-mediated processes occurring in association with the tumor [41]. All of this highlights the heterogeneous nature of NSCLC and emphasizes the importance of correlating clinical manifestations with imaging and pathological studies for timely diagnosis and effective management of the disease.

### 5.1. Respiratory Symptoms

Respiratory manifestations are the most common clinical features of NSCLC and are often the initial reason for patient presentation. Cough is the dominant symptom and may present in various forms—persistent, dry, or productive—depending on the degree of bronchial irritation and airflow obstruction. In central tumors, cough usually occurs early because the lesion involves large airways, stimulating cough receptors or causing partial narrowing of the bronchi. Hemoptysis, although uncommon, usually reflects erosion of the bronchial vasculature, friability of the tumor surface, or bleeding from necrotic intratumoral tissue. Even minor hemoptysis requires careful evaluation because it may indicate aggressive local invasion [42]. Dyspnea develops either from segmental or lobar bronchial obstruction, which can lead to distal atelectasis, impairing ventilation, or from tumor-induced pleural effusion or compression of the lung parenchyma, which reduces the effective respiratory surface area, resulting in respiratory distress. In advanced stages, tumor expansion or post-obstructive changes may further compromise pulmonary reserve [43]. Recurrent or persistent respiratory infections are another important manifestation, especially when a tumor obstructs the bronchial lumen. Impaired airflow promotes mucus retention, reduces mucociliary clearance, and creates an environment conducive to bacterial growth. These infections—which usually present as repeated episodes of pneumonia or chronic bronchitis—may have a limited response to standard antibiotic therapy. In some patients, such infectious episodes serve as the earliest clue suggesting an underlying endobronchial malignancy [44].

### 5.2. General Symptoms

In addition to respiratory manifestations, patients with NSCLC also present with general, nonspecific symptoms. Among these, unintentional weight loss is notable, serving as a clinical indicator of active metabolism and tumor progression. This weight loss often coincides with anorexia, alterations in nutrient absorption and utilization, and the development of cachexia, a complex metabolic syndrome characterized by muscle atrophy, fat depletion, and systemic inflammation [45]. Persistent fatigue is another common feature and may result from multiple overlapping mechanisms. Chronic inflammation induced by tumor-secreted cytokines, anemia secondary to bone marrow suppression or chronic disease, and the direct metabolic demands of tumor growth contribute to the depletion of energy reserves. Functional impairment caused by respiratory compromise, pain, or reduced exercise tolerance further exacerbates the feeling of fatigue. Although these general symptoms are nonspecific and can be attributed to a wide range of chronic conditions, their presence in combination—or in the context of progressive respiratory disease—may signal an underlying neoplastic process. Over time, these systemic manifestations contribute to the overall deterioration of health, often prompting patients to seek medical evaluation and requiring further diagnostic investigations. Therefore, awareness of these subtle but impactful signs is essential for early detection and comprehensive management of NSCLC [46].

### 5.3. Paraneoplastic Syndromes

Paraneoplastic syndromes are systemic disorders that arise from the ectopic production of hormones, cytokines, or growth factors by tumor cells. These syndromes, although relatively rare, can serve as early diagnostic indicators of NSCLC [47]. Hypercalcemia occurs primarily in association with squamous cell carcinoma, and tumor cells may produce parathyroid hormone-related protein, leading to elevated serum calcium levels. Clinically, it may present with nausea, vomiting, polyuria, dehydration, confusion, and, in severe cases, cardiac arrhythmias or neurological manifestations, occasionally representing a medical emergency requiring rapid intervention [48]. Although ectopic hormone secretion, such as ACTH production, is more commonly associated with small-cell lung cancer, it can occasionally occur in NSCLC. In these rare cases, patients may develop paraneoplastic manifestations, including features of Cushing’s syndrome and hyperpigmentation, reflecting the systemic impact of aberrant hormone production. While uncommon, recognition of these phenomena in NSCLC underscores the importance of comprehensive clinical evaluation and may influence both diagnostic workup and management strategies [49]. Another notable paraneoplastic phenomenon is hypertrophic osteoarthropathy, characterized by periostitis of the digits and joint pain. This syndrome is more commonly associated with adenocarcinoma, and although its precise pathophysiological mechanisms remain incompletely elucidated, it is hypothesized to involve circulating growth factors or vascular endothelial mediators that stimulate the formation of new bone and soft tissue [50]. In addition, there is the syndrome of inappropriate antidiuretic hormone secretion, which causes hyponatremia, skin hyperpigmentation such as acanthosis nigricans, and paraneoplastic coagulopathy characterized by blood hypercoagulability and the development of thrombosis. Early recognition of these syndromes not only facilitates timely diagnostic investigations but may also improve the likelihood of identifying NSCLC at a stage that may be amenable to curative or disease-modifying treatment.

## 6. Paraclinical Evaluation

Paraclinical assessment of NSCLC is an essential element in diagnosis, influencing both staging and selection of appropriate therapeutic strategies. This evaluation involves advanced imaging modalities, targeted invasive procedures for tissue acquisition, and increasingly modern molecular analyses. Imaging methods provide critical information about tumor location, size, local invasion, and the presence of nodal or distant metastases. Posterior–anterior chest radiography, computed tomography (CT), and magnetic resonance imaging (MRI) allow for detailed anatomical mapping, while positron emission tomography (PET) and PET/CT fusion imaging provide functional information, such as metabolic activity, that can improve the detection of occult metastatic disease and guide biopsy targeting. Tissue sampling remains the key to definitive histopathological diagnosis. Techniques range from minimally invasive procedures—such as flexible bronchoscopy with biopsy, ultrasound-guided endobronchial fine-needle aspiration (EBUS-FNA), and CT-guided transthoracic biopsy—to surgical approaches, including mediastinoscopy or video-assisted thoracoscopic surgery (VATS) for more extensive sampling. These methods enable precise classification of tumor subtypes and assessment of histological features critical for prognosis. Advances in molecular and genomic profiling have transformed the clinical landscape. Analysis of driver mutations, genetic rearrangements, and predictive biomarkers informs the use of targeted therapies and immunotherapeutic agents, enabling a shift from generalized to personalized oncology management. Techniques such as next-generation sequencing (NGS) and circulating tumor DNA (ctDNA) assays further refine the diagnostic process by detecting actionable alterations and monitoring minimal residual disease [51].

### 6.1. Imaging

Chest radiography remains one of the oldest and most widely used diagnostic tools in the evaluation of patients with suspected pulmonary disease, including those ultimately diagnosed with NSCLC. Although its diagnostic performance is limited compared with more advanced imaging modalities, it continues to serve as a practical and widely available initial evaluation technique, particularly in symptomatic individuals or in situations where rapid triage is required. Although it has relatively low sensitivity, chest radiography can reveal many abnormalities that require further investigation. These findings may include pulmonary nodules or masses, areas of lobar or segmental atelectasis, pleural effusions, or mediastinal widening, each of which may raise suspicion of an underlying neoplastic process [52]. Radiographs are particularly useful for detecting large peripheral tumors or secondary changes, such as atelectasis caused by endobronchial obstruction. However, the value of chest radiography is limited by several disadvantages. Small lesions, especially those <1 cm, often escape detection, and centrally located tumors may be obscured by overlapping anatomical structures, such as the heart or major vessels. Dense and overlapping thoracic structures may further mask early-stage disease, reducing the sensitivity of radiographs for detecting subtle or early malignancies [52]. Despite these shortcomings, chest radiography remains clinically relevant. It provides a rapid, inexpensive, and widely available means of identifying macroscopic abnormalities and guiding the selection of more sensitive imaging modalities, such as chest CT or PET-CT. Contrast-enhanced computed tomography of the chest is widely considered the cornerstone imaging modality in the diagnosis of NSCLC. Its high spatial resolution and ability to precisely delineate thoracic anatomy make it indispensable for the characterization of both primary tumors and associated structural involvement. By administering intravenous contrast, chest CT enhances visualization of vascular, mediastinal, and pleural structures, allowing for a more precise assessment of tumor size, contour, and internal features. This level of detail facilitates the assessment of contact with adjacent anatomical structures, such as invasion of pulmonary arteries, veins, bronchi, and the chest wall [53]. In addition, it reliably identifies pleural infiltration, satellite lesions, and mediastinal or hilar lymphadenopathy, all of which have significant staging implications. Beyond its role in tumor characterization, chest CT is fundamental in differential diagnosis, helping to distinguish malignant lesions from benign entities such as infections, granulomatous disease, or vascular abnormalities. It is also essential in planning invasive procedures, including percutaneous biopsies and endobronchial sampling, guiding physicians to the most accessible and diagnostically significant ones. In the context of staging, it helps determine tumor extension and regional lymph node involvement within the TNM classification system, and it also directly informs therapeutic decision-making, influencing eligibility for surgical resection, radiotherapy planning, and selection of systemic treatments [53]. PET-CT, most often performed with fluorodeoxyglucose, has become the standard imaging technique for assessing the metabolic activity of tissues suspected of malignancy. It is particularly valuable in detecting regional lymph node involvement and distant metastatic spread, areas in which it consistently demonstrates greater sensitivity than standard chest CT [54]. The increased glucose uptake characteristic of most malignant cells allows it to highlight metastatic deposits that may be morphologically incongruent or occult on structural imaging. PET-CT can also reveal synchronous primary tumors, atypically localized metastatic disease, or early recurrences, thus influencing treatment planning and monitoring strategies [54]. Magnetic resonance imaging (MRI) of the brain plays a critical role in the evaluation of patients with non-small-cell lung cancer when there is clinical suspicion of brain involvement. Due to its superior soft tissue contrast and high sensitivity, brain MRI remains the most accurate modality for identifying brain metastases, especially small lesions or those located in anatomically complex regions that may be missed by computed tomography. Brain MRI is strongly recommended for symptomatic individuals, for example, those presenting with headache, neurological deficits, seizures, or cognitive impairment, as well as for patients with advanced disease, in whom the likelihood of occult brain metastases is increased [55]. In certain histologic subtypes, particularly adenocarcinoma, brain MRI may be warranted even in the absence of neurological symptoms, as brain metastases are common in this histologic subtype. Identification of intracranial metastases can modify systemic treatment plans, determine eligibility for targeted therapies or immunotherapy, and guide the selection between stereotactic radiotherapy and whole-brain radiotherapy [55]. Cardiac magnetic resonance imaging (CMR) has proven to be the most sensitive and specific method for detecting cardiac metastases, providing precise information on lesion location, size, extent, and involvement of adjacent structures. Compared with CT or echocardiography, CMR offers high-resolution, multiplanar assessment and tissue characterization, making it the gold standard for identifying these metastases. Integrating CMR into the diagnostic workflow for advanced NSCLC can help explain unexplained systemic symptoms and guide personalized therapeutic strategies, thereby improving prognosis for patients with metastatic disease [56].

### 6.2. Histological and Cytological Diagnosis

Diagnostic certainty in NSCLC is based on histological or cytological confirmation, which remains the gold standard for identifying the specific tumor subtype. Obtaining a sufficient volume of high-quality biological material is essential—not only for accurate morphological evaluation but also to allow immunohistochemistry, which has become indispensable for guiding personalized therapeutic strategies in modern oncology. The choice of sampling technique depends on the location of the tumor, the patient’s characteristics, and the need for auxiliary methods. Flexible bronchoscopy is the first-line diagnostic procedure for centrally located tumors, due to its ability to provide direct endoscopic visualization of the tracheobronchial tree. This technique facilitates targeted biopsy of endobronchial lesions while allowing for bronchial aspirate collection, brush cytology, and transbronchial biopsy of adjacent parenchymal regions, when necessary. Its main advantage lies in the minimally invasive access to the central airways and the possibility of obtaining multiple tissue fragments, thus increasing diagnostic accuracy [57]. A diagnostic method that also contributes to the staging of lung cancer is ultrasound-guided transbronchial fine-needle aspiration (EBUS-TBNA). This technique is used especially in cases where the tumor is not accessible by fiberoptic bronchoscopy or does not show endobronchial expression, and there is tumor infiltration in the mediastinal nodes or, in the case of inconclusive biopsies, is performed directly from the endobronchial lesion [57]. For peripheral pulmonary nodules or masses that cannot be reached endoscopically, CT-guided transthoracic needle biopsy is considered the procedure of choice. This method ensures a high diagnostic yield due to its precise imaging-based guidance, which enables accurate targeting of even small or deep-seated lesions. Despite its effectiveness, the procedure carries a recognized risk of complications, most commonly pneumothorax, necessitating careful patient selection and performance in centers with substantial experience. Specimens obtained through transthoracic biopsy are typically suitable for comprehensive histologic examination and molecular testing, including mutation analysis and immunohistochemical profiling [58]. When less invasive diagnostic approaches fail to provide conclusive results, video-assisted thoracoscopic surgery (VATS) represents an advanced alternative that allows direct visualization and sampling of pulmonary nodules, pleural lesions, or mediastinal lymph nodes. VATS provides access to high-quality tissue samples, improving the likelihood of establishing an accurate diagnosis. In addition, surgical specimens obtained by lobectomy, segmentectomy, or other forms of lung resection constitute the most complete material for histopathological evaluation, immunohistochemistry, and molecular analyses [59]. These samples play a crucial role in accurate pathological staging, which is essential for determining prognosis and selecting the optimal therapeutic course.

### 6.3. Molecular and Immunohistochemical Analyses

The integration of molecular and IHC analyses has become the cornerstone of contemporary NSCLC management, moving beyond morphology-based classification to a precision oncology framework. Histopathological evaluation, while essential, is now complemented by IHC markers and comprehensive genomic profiling to guide diagnostic confirmation, prognostic stratification, and therapeutic selection. Molecular testing is considered mandatory in adenocarcinoma and strongly recommended in poorly differentiated or non-squamous NSCLC, given the high probability of identifying oncogenic factors with therapeutic implications [60]. A comprehensive genomic profile is essential to guide precision therapy in adenocarcinoma and in subtypes of NSCLC that do not show clear squamous differentiation. Adenocarcinomas arise from epithelial cells in the terminal respiratory unit and have variable morphological appearances, being of several acinar, lepidic, and papillary types, usually expressing thyroid transcription factor 1 (TTF1), Napsin A and cytokeratin 7 (CK7) [61]. There is some data in the literature suggesting that in Caucasian patients, the lack of TTF1 expression by tumor cells has a strong negative predictive value for activating EGFR mutations in adenocarcinomas, although this is less clear for ALK/ROS1. On the other hand, squamous carcinomas arise from basal bronchial epithelial cells, and morphologically, these lesions usually show keratinization and intercellular bridges with concomitant expression of p40, p63 and cytokeratin 5/6. In this context, testing for EGFR mutations, ALK and ROS1 rearrangements, and other key genomic alterations such as KRAS, BRAF, or MET mutations has become standard practice. In large cell lung cancer, key IHC markers are synaptophysin or chromogranin A, and in this context, multiple actionable genetic alterations like EGFR, ALK, KRAS, and ROS1 are tested. In the case of adenocarcinomas in “non-smokers”, they present a significantly higher frequency of mutations in EGFR, ALK, and ROS, while smokers present a high frequency of KRAS mutations, suggesting potentially different pathogenetic mechanisms of tumor development in smokers and non-smokers. The detection of these molecular abnormalities enables the use of tyrosine kinase inhibitors (TKIs) and other targeted agents, which have demonstrated superior response rates, improved quality of life, and significantly reduced systemic toxicity compared with traditional cytotoxic chemotherapy. As a result, molecular stratification transforms the therapeutic landscape, offering individualized treatment pathways tailored to the tumor’s specific genetic profile [62]. Assessment of PD-L1 expression through immunohistochemistry has emerged as a cornerstone of immunotherapy selection in NSCLC. PD-L1 levels are reported as the tumor proportion score (TPS), reflecting the percentage of tumor cells exhibiting membranous staining. This quantitative evaluation guides the decision to initiate immune checkpoint inhibitor therapy, either as monotherapy in cases with high PD-L1 expression or in combination with chemotherapy when moderate or heterogeneous expression patterns are observed. Although PD-L1 is not an ideal biomarker—owing to intratumoral heterogeneity, variability in testing platforms, and limited predictive accuracy—it remains the most widely implemented tool for determining eligibility for immunotherapy and for estimating the likelihood of therapeutic response [63]. Molecular alterations are not merely academic; they are clinically actionable. Identification of targetable mutations directly informs the use of tyrosine kinase inhibitors, BRAF/MEK inhibitors, and immune checkpoint inhibitors, providing superior response rates, improved survival, and effective systemic and intracranial disease control. Consequently, comprehensive molecular and IHC profiling is now considered mandatory for all newly diagnosed advanced NSCLC cases, representing a shift from morphology-centric to biomarker-driven decision-making. Beyond guiding treatment, integrated analyses refine prognostic assessment. Certain histologic patterns combined with specific molecular alterations can predict disease aggressiveness, recurrence risk, and likelihood of response to immunotherapy, enabling truly individualized patient management.

## 7. Treatment

**Figure 2:**
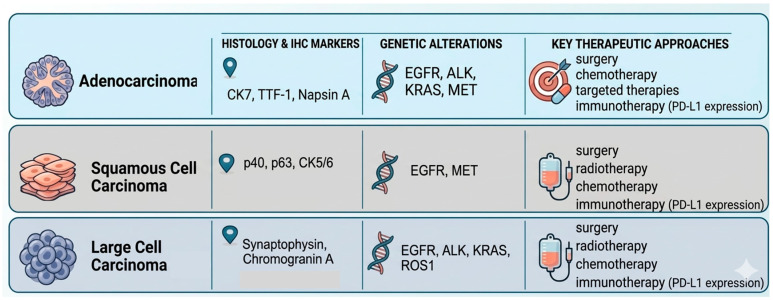
From Histopathological Classification to Precision Oncology.

The treatment of NSCLC results from the integration of clinical, imaging, histological, and molecular information in a multidisciplinary context involving a pulmonologist, an oncologist, a pathologist, and a thoracic surgeon. Treatment selection is no longer stage-based alone, but is critically dependent on molecular and immunohistochemical profiling. (Figure 2) [64]. In localized stages, curative treatment is pursued, while in advanced stages, the goals are to prolong survival and improve quality of life. Stages I–II are generally managed with curative intent by surgery or stereotactic radiotherapy, while stage III involves sequential or concomitant combinations of surgery, radiotherapy, and chemotherapy [65,66]. Stage IV is managed mainly by systemic therapy, guided by molecular and immunological profiling [67]. Evaluation of biomarkers such as EGFR, ALK, ROS1, BRAF, KRAS, or PD-L1 expression is indispensable before initiating treatment in most cases of advanced disease [68].

### 7.1. Surgical Treatment

Surgical resection remains the standard treatment with curative intent for early-stage (I–II) and selected stage IIIA NSCLC with limited and resectable nodal involvement. The primary goal is complete excision of the tumor with negative margins while preserving as much functional lung parenchyma as possible. Lobectomy is considered the gold standard, providing an optimal balance between oncological control and preservation of lung function. Sublobar resections, such as anatomical segmentectomy, may be indicated for small peripheral tumors (<2 cm) or in patients with significant comorbidities. Pneumonectomy is reserved for cases in which limited resections cannot achieve complete tumor clearance, due to its higher morbidity [66]. Adjuvant chemotherapy is recommended in the presence of nodal involvement or other high-risk features. Furthermore, in patients with EGFR sensitizing mutations, adjuvant therapy with EGFR tyrosine kinase inhibitors—particularly osimertinib—significantly reduces the risk of recurrence and improves disease-free survival [69].

### 7.2. Radiotherapy

Radiotherapy plays a key role in the management of NSCLC as a curative, definitive or palliative treatment modality, depending on the stage of the disease, the characteristics of the tumor, and the operability of the patient. By minimizing radiation-induced toxicity to surrounding normal tissues, various radiation techniques have distinguished and evolved. In the case of patients with early-stage NSCLC who are medically inoperable or refuse surgery, stereotactic body radiotherapy has become the treatment of choice. It offers the possibility of administering high doses of radiation in a limited number of fractions with maximum precision, resulting in excellent local control rates, comparable to those obtained with surgical resection. This approach is very effective for small peripheral tumors, with a diameter of less than 3 cm, where the risk of toxicity to critical thoracic structures is relatively low. For patients with locally advanced disease, radiotherapy is most often administered in combination with systemic chemotherapy [70]. Concurrent chemoradiotherapy is the standard of care for inoperable stage III NSCLC and has demonstrated superior survival outcomes compared with sequential treatment approaches. The cumulative effect of chemotherapy increases radiosensitivity, thereby improving local tumor control, although it is associated with an increased risk of acute and late toxicities, requiring careful patient selection and monitoring [71]. Technological innovations in radiotherapy have further optimized the precision and safety of treatment. Techniques such as intensity-modulated radiotherapy and image-guided radiotherapy allow for increased tumor dose while sparing adjacent organs at risk, including the lungs, heart, and esophagus. This has reduced treatment-related morbidity and improved patient tolerance to treatment. In addition to its curative and definitive applications, radiotherapy remains an essential component of palliative care in NSCLC, being effective in alleviating tumor-related symptoms such as pain, hemoptysis, dyspnea, and complications arising from mediastinal compression, thus improving the quality of life in patients with advanced or metastatic disease [70].

### 7.3. Chemotherapy

Chemotherapy continues to be a fundamental component of the therapeutic strategy for NSCLC, especially in patients with advanced disease and as adjuvant or neoadjuvant treatment in early stages, and remains indispensable in multiple clinical settings. Platinum-based doublet chemotherapy constitutes the basis of systemic treatment for NSCLC. Standard regimens usually combine cisplatin or carboplatin with a third-generation cytotoxic agent such as vinorelbine, gemcitabine, paclitaxel, or pemetrexed. Chemotherapeutic agents are chosen according to the patient’s general condition, comorbidities, and histological subtype. Pemetrexed has demonstrated superior efficacy and a more favorable toxicity profile, especially in adenocarcinoma, and is therefore preferentially recommended in this subgroup, while its use is contraindicated in squamous cell carcinoma due to inferior results [72]. In inoperable stage III NSCLC, concurrent chemoradiotherapy is the standard of care, providing improved survival compared with sequential therapeutic approaches. In addition, consolidation therapy with the anti-PD-L1 monoclonal antibody durvalumab, after definitive chemoradiotherapy, has been shown to significantly improve progression-free survival and overall survival, establishing a new therapeutic paradigm in this setting [73]. In metastatic disease (stage IV), chemotherapy is used either alone or in combination with immunotherapy, depending on tumor PD-L1 expression. In the absence of actionable oncogenic driver mutations, chemoimmunotherapy combinations have demonstrated superior clinical outcomes compared with chemotherapy alone [74]. Conversely, in patients with high PD-L1 expression, immunotherapy-based therapeutic regimens may be preferred, with chemotherapy reserved for cases of disease progression or contraindications to immunotherapy [74].

### 7.4. Targeted Therapy

Targeted therapy represents one of the most significant advances in the management of NSCLC, reflecting a paradigm shift toward precision oncology based on the identification of oncogenic driver alterations. By selectively inhibiting molecular pathways essential for tumor growth and survival, targeted agents have demonstrated superior efficacy and improved tolerability compared with conventional chemotherapy in appropriately selected patient populations [75]. Among the most extensively studied targets in NSCLC are activating mutations of the epidermal growth factor receptor (EGFR), which are predominantly observed in adenocarcinoma histology. First-, second-, and third-generation EGFR tyrosine kinase inhibitors (TKIs), including erlotinib, gefitinib, afatinib, and osimertinib, have shown substantial clinical benefit in tumors harboring sensitizing EGFR mutations, particularly exon 19 deletions and the L858R point mutation. Osimertinib is currently the recommended first-line treatment because it works better, has fewer side effects, and can better penetrate the central nervous system. This leads to better control of the disease throughout the body and in the brain [76]. Another specific group of non-small-cell lung cancer (NSCLC) is defined by changes in the anaplastic lymphoma kinase (ALK) gene. ALK inhibitors, such as alectinib, brigatinib, and lorlatinib, have shown high response rates and lasting disease control in tumors with ALK mutations. These drugs are particularly effective at preventing and treating brain metastases, which are common in this group of patients [77]. Tumors harboring ROS1 gene fusions may be effectively treated with TKIs such as crizotinib and entrectinib, both of which have shown meaningful systemic and intracranial activity [78]. In patients with BRAF V600E mutations, combined inhibition of BRAF and MEK using dabrafenib in conjunction with trametinib has emerged as the standard targeted approach, yielding significant clinical responses [79]. More recently, the development of KRAS G12C inhibitors, including sotorasib and adagrasib, has addressed one of the most common cancer-causing factors in NSCLC, which was previously considered impossible to treat with drugs [80]. These treatments have shown promising results in patients with advanced disease who have not responded to previous systemic therapies, thus expanding the treatment options for this common molecular subtype. Accurate identification of targetable molecular changes is crucial for the best use of targeted therapy. Therefore, comprehensive molecular profiling using next-generation sequencing (NGS) is strongly recommended whenever possible, as it allows for the simultaneous detection of multiple genomic alterations and supports personalized treatment choices [75].

### 7.5. Immunotherapy

Immunotherapy is notable for providing significant and sustained improvements in survival outcomes with immune checkpoint inhibitors. These agents inhibit key immune checkpoint pathways that tumors use to evade immune surveillance, and the most widely used inhibitors target programmed cell death protein 1 (PD-1) and its ligand PD-L1. Monoclonal antibodies directed against PD-1, such as pembrolizumab and nivolumab, as well as PD-L1 inhibitors, including atezolizumab and durvalumab, have demonstrated robust clinical efficacy across multiple disease stages. These agents stimulate T-cell activation and proliferation, thereby promoting immune-mediated tumor cell killing by blocking the PD-1/PD-L1 interaction. Pembrolizumab monotherapy is recommended for patients whose tumors have high PD-L1 expression (tumor proportion score ≥ 50%) in the absence of actionable molecular alterations and in the first-line treatment of metastatic NSCLC. In this subgroup, immunotherapy alone has been shown to significantly improve overall survival and quality of life compared with platinum-based chemotherapy [81]. For patients with low or absent PD-L1 expression, immune checkpoint inhibitors are most commonly administered in combination with chemotherapy. Chemoimmunotherapy regimens have demonstrated significant survival benefits regardless of PD-L1 status and have therefore become the standard of care in this clinical setting [82]. In unresectable stage III NSCLC, consolidation immunotherapy with durvalumab after definitive concurrent chemoradiotherapy is the current standard of care. This strategy has been associated with durable improvements in progression-free survival and overall survival, marking a major advance in the management of locally advanced disease [83]. A defining feature of immunotherapy is the potential for durable and long-lasting responses. A subgroup of patients achieves prolonged disease control or complete remission, highlighting the ability of immune checkpoint inhibition to induce sustained antitumor immunity and fundamentally alter the natural history of advanced NSCLC.

### 7.6. Palliative Treatment

In patients with advanced NSCLC or those with limited functional reserve who are not candidates for curative intent therapies, the main goals of treatment include symptom control, optimization of quality of life, and psychosocial support. Therefore, palliative care is an essential component of the complex management of NSCLC and should be individualized according to the severity of symptoms and patient preferences. Effective pain management through appropriate analgesic therapy with opioid and non-opioid medications is fundamental to improving patient comfort. Dyspnea, one of the most common and distressing symptoms, can be managed primarily by supplemental oxygen therapy when indicated and by non-pharmacological interventions [84]. Recurrent malignant pleural effusions can be addressed by therapeutic thoracentesis, indwelling pleural catheters, or pleurodesis, depending on the context. In addition, prompt treatment of infectious complications and provision of adequate nutritional support are crucial in maintaining functional status and reducing symptom burden [84]. Palliative radiotherapy can provide rapid and effective relief of bone pain, refractory cough, hemoptysis, and symptoms related to compression of mediastinal or neural structures, thereby contributing significantly to symptom relief and overall well-being [85]. Importantly, early integration of palliative care alongside disease-directed treatment has demonstrated substantial benefits in patients with metastatic NSCLC. Early palliative involvement is associated with improved symptom control, increased quality of life, better patient and carer satisfaction, and, in some studies, prolonged survival. This integrated approach emphasizes the importance of incorporating palliative principles throughout the disease trajectory, rather than reserving them exclusively for end-of-life care [84].

## 8. Prognosis

The prognosis of this type of cancer is influenced by clinical, histological, molecular, and therapeutic factors, with the stage of the disease at diagnosis remaining the most important determinant of prognosis. Overall survival is significantly superior in early stages of the disease, where complete surgical resection with curative intent is feasible, compared with advanced stages, where regional lymph node involvement or distant metastases preclude definitive local treatment [86]. Histological subtype also plays a significant role in prognostic stratification, with adenocarcinoma being the most common histological variant of NSCLC and generally associated with a poor prognosis. However, outcomes have improved substantially due to the availability of targeted therapies and immunotherapy in molecularly tested patients. Squamous cell carcinoma, on the other hand, often presents a more aggressive clinical course and is associated with a more limited spectrum of actionable molecular alterations, which may restrict therapeutic options. Large cell carcinoma, due to its biological heterogeneity and predisposition to early dissemination, is usually associated with a poor prognosis and high metastatic potential [87]. The presence of actionable oncogenic factors, such as activating mutations of EGFR or rearrangements of the ALK and ROS1 genes, allows the use of targeted therapies that are associated with higher response rates, prolonged progression-free survival, and improved overall survival. Similarly, increased PD-L1 expression has been correlated with increased responsiveness to immune checkpoint inhibitors [88]. In contrast, molecular alterations, such as TP53 mutations or complex genomic profiles, are frequently associated with more aggressive tumor behavior, resistance to therapy, and poorer clinical outcomes [89]. Survival outcomes vary considerably depending on the stage of the disease. In patients with stage I NSCLC, five-year survival rates can exceed 60–70%, reflecting the effectiveness of surgical treatment in early-stage disease. In stage II, five-year survival rates drop to approximately 40–50%, while in stage III disease, long-term survival is observed in only 20–30% of patients, despite multimodal therapeutic approaches [90]. In metastatic stage IV NSCLC, median overall survival typically ranges from 12 to 30 months, depending on tumor biology, molecular profile, and access to contemporary systemic therapies, including targeted agents and immunotherapy [91]. Ultimately, response to treatment, durability of disease control, and the ability to tailor therapy to individual tumor characteristics remain essential for improving the prognosis of patients with NSCLC. Continued advances in molecular diagnostics and therapeutic innovation are expected to further refine prognostic assessment and improve survival outcomes in the future.

## 9. Conclusions

The landscape of NSCLC has evolved far beyond traditional histopathological classification. While histology remains a fundamental component of diagnosis, it is no longer sufficient to guide optimal patient management in the era of precision oncology. Comprehensive molecular profiling has become the standard of care, enabling the identification of clinically actionable alterations and guiding targeted therapeutic strategies. The integration of histological, immunohistochemical, and genomic data is essential for accurate diagnosis, prognostic assessment, and individualized treatment planning. Looking ahead, the future of NSCLC management lies in combining innovative targeted therapies with immunotherapies and incorporating emerging biomarkers to further refine patient selection and improve outcomes, underscoring the necessity of a holistic, data-driven approach that bridges morphology with molecular precision.

## Data Availability

The data presented in this study are available on request from the corresponding authors.

## Abbreviations

The following abbreviations are used in this manuscript:

NSCLC	Non-small-cell lung cancer
SCLC	Small-cell lung cancer
EGFR	Epidermal growth factor receptor
ALK	Anaplastic lymphoma kinase
ROS1	Proto-oncogene tyrosine-protein kinase ROS
KRAS	Kristen rat sarcoma viral oncogene homolog
PD-1	Programmed cell death protein
PD-L1	Programmed death-ligand 1
PM2.5	Fine particles with diameters less than 2.5 μm
DNA	Deoxyribonucleic acid
PI3K/AKT	Phosphatidylinositol 3-kinase/protein kinase B
JAK/STAT	Janus kinases/signal transducer and activator of transcription proteins
FGFR1	Fibroblast growth factor receptor 1
TNM	Tumor-node-metastasis
ACTH	Adrenocorticotropic hormone
MRI	Magnetic resonance imaging
CT	Computed tomography
PET	Positron emission tomography
EBUS-TBNA	Endobronchial ultrasound-guided transbronchial needle aspiration
VATS	Video-assisted thoracoscopic surgery
NGS	Next-generation sequencing
ctDNA	Circulating tumor DNA
TTF1	Thyroid transcription factor 1
TKIs	Tyrosine kinase inhibitors
TP53	Tumor protein 53
TPS	Tumor proportion score
